# Study on the preparation and mechanical properties of purple ceramics

**DOI:** 10.1038/s41598-023-35957-0

**Published:** 2023-05-30

**Authors:** Lihe Wang, Yonghui Wang, Qingchun Wang, Yuwei Ma, Fei Ruan, Yonghe Zhang, Haodong Lv, Qiang Jing, Jinxiao Bao

**Affiliations:** Inner Mongolia Key Laboratory of Advanced Ceramics and Device, School of Materials and Metallurgy, Inner Mongolia University of Science and Technology, Baotou, 014010 Inner Mongolia China

**Keywords:** Materials science, Nanoscience and technology

## Abstract

This paper aims at preparing a smart wearable purple ceramic that meet the color requirements of purple smart wear in the market after using zirconate neodymium as a chromogenic agent. However, the mechanical performance of zirconate neodymium purple ceramic is not satisfactory, especially it has an extremely low fracture toughness. To solve this, a 3 mol% yttria-stabilized zirconia (3YSZ) is added to zirconate neodymium in the preparation of multiphase ceramics to improve its mechanical properties. In this experiment, a series of ceramic samples with addition of increasing amount of 3YSZ 0, 20, 40, 50, 60, 70 and 80% were prepared in the 1400–1500 °C sintering temperature range. It was found that at the same temperature, the mechanical properties of the ceramic samples gradually improved with the increase in the 3YSZ content. Moreover, with the same content, the mechanical properties of the ceramic samples gradually improved with the decrease in temperature. The results show that when 3YSZ has a mass fraction of 80% and is sintered at 1400 °C, the fracture toughness of the prepared ceramic samples reaches 8.15 MPa‧m^1/2^, which is nearly two times higher than that of the monolithic neodymium zirconate 2.57 MPa‧m^1/2^. The Vickers hardness of the prepared ceramic samples reached 12.93 GPa, which is nearly 88% higher than the undoped neodymium zirconate. This indicates that the samples can be applied in smart wearables, such as mobile phone backplane, with a certain practical significance for engineering toughening of zirconate ceramics.

## Introduction

Colored ceramics are widely used in various industries with the rapid development of science and information technology. As colored ceramics are bright in color, have a good metallic texture, and mainly have low signal interference characteristics, they have begun to be increasingly used in smart wearables such as mobile phone backplanes, watch rings, and others. However, brittleness is a major weakness of these ceramics, especially neodymium zirconate ceramics; therefore, improving the mechanical properties, especially the fracture toughness of the ceramics, has become a top priority. Matsumura et al.^1^ prepared a La_2_Zr_2_O_7_ ceramic material using the hydenzine method and measured that its fracture toughness and bending strength reached 1.9 MPam^1/2^ and 172 MPa, respectively. Lee et al.^2^ added yttrium oxide to gadolinium zirconate to prepare composite ceramics and found that the Vickers hardness, increased from 6 to 10 GPa. Yu Zhang et al.^3^ doped ytterbium oxide-stabilized zirconia with gadolinium zirconate, resulting in significantly improved fracture toughness of the material.

Zirconia has the characteristics of phase change toughening; therefore, martensitic phase change is one of its major characteristic. Hence, it is commonly used to improve the fracture toughness of ceramic materials^4^. Zirconia has been extensively studied, and research on zirconia ceramics remains a hotspot. Zirconia ceramics are increasingly widely used in the aerospace, electronics, metallurgy, and communications, among other industries^5^. Yttrium dioxide is added to most zirconia. In particular, 6–8 mol%Y_2_O_3_ is added to zirconia to obtain a cubic phase, and 3–6 mol% yttrium is added to zirconia to obtain a partially stabilized zirconia. A fully stable zirconia has a high ionic conductivity, and a partially stabilized zirconia has excellent mechanical properties at room temperature and high temperature^6^.

Many transition metals do not have only one valence state in the compound but also coexist in multiple valence states. Fujimori^7^ called this phenomenon the theory of mixed valence state. This is due to the existence of point defects in the crystal, resulting in the crystal having special magnetic, optical, and electrical properties. So, far, there are red zirconia ceramics in the market, which are prepared by adding iron trioxide to zirconia to produce the red color^8^. Purple ceramics are prepared by adding neodymium oxide or neodymium zirconate to zirconia^9^. Sky-blue ceramics are prepared by adding nickel oxide, and alumina oxide to zirconia^10^. Green ceramics are prepared by adding a certain amount of nickel oxide , alumina oxide, chromium oxide and silicon oxide to zirconia^11^. The colored ceramics prepared in these markets have a wide range of applications, primarily in decorations and smart wearables, such as mobile phone backplanes, watch rings, and others^12,13^.

In this study, 3YSZ was added to neodymium zirconate to prepare composite ceramics, which can not only meet the color requirements of intelligence wear purple ceramics but also improve the fracture toughness of neodymium zirconate purple ceramics to enable a safe processing without premature catastrophic failure.

## Materials and methods

### Sample preparation

In this experiment, the solid-phase ball milling method was used to prepare the ceramic samples. Here, 3YSZ (ZrO_2_: purity ≥ 99.9%, Decheng Chemical Co., Ltd.; Y_2_O_3_: purity ≥ 99.9%, Yuekai Metal Materials Co., Ltd.) was added to neodymium zirconate (purity ≥ 99.9%, Yuekai Metal Materials Co., Ltd.) for high-energy ball milling for 24 h at 450 rpm and with ball-to-powder weigth ratio of 4:1. Milling media were in Ethanol absolute (CH_3_CH_2_OH: purity ≥ 99.7). The mixed slurry was dried at 338 K for 24 h and was then ground with a mortar. After grinding, it was sieved with an 80 mesh sieve. Afterward, approximately 3 g of the sieved powder was placed in a mold with a diameter of 25 mm. A tabletop electric tablet press was used for shaping 25 mm diameter pellets, with an applied pressure of 8 MPa. A cold isostatic press with a pressure of 200 MPa was then used before sintering at the set temperature for 3 h in an air furnace (Hefei Kejing Material Technology Co., Ltd, KSL-1700X-A2).

In this work, a series of ceramic samples with 3YSZ mass fractions of 0, 20, 40, 50, 60, 70 and 80% were prepared at sintering temperatures of 1500 °C, 1450 °C, and 1400 °C.

### Characterization

According to the Bragg formula, X-ray diffraction (XRD) was used to study the crystalline phase. The instrument diffraction target for the Cu target, and the working voltage and current were 40 kV and 40 mA, respectively. The scanning range was 20 ~ 80° (2θ), and the scanning speed 2°/min. The phase composition and the related cell parameters were obtained by comparing with the standard card through the Jade software.

Field emission scanning electron microscopy (FE-SEM, Zeiss, Sigma 500) was used to analyse the surface morphology of the prepared ceramic samples, mainly using secondary electron imaging. The surface of all the samples was fine polished by an automatic polishing machine (Shenyang Kejing Automation Equipment Co., Ltd, UNIPOL-1200S) and then hot etched in a high-temperature furnace. The hot etching temperature was set at 1400 °C for 20 min, and the high-temperature hot rotting was necessary to remove the residual stress and scratches on the sample surface during the fine polishing.

An automatic Vickers hardness tester (MATSUZAWA in Japan, Via-S) was used, with a 2 kgf load held for 10 s. Before the determination of the ceramic sample surface polishing treatment to observe the test sample in the microscope with no obvious scratches. All the samples were annealed at 1300 °C for half an hour. This operation was performed to eliminate the residual stresses generated during polishing and grinding. The Vickers hardness of the samples is measured by a Vickers microhardness tester (VIA-S). All ceramic samples were tested at least five times. In this experiment, the load and the pressure holding time are 2000 GF (9.8 N) and 10 s, respectively. The specific calculation formula is as follows:

1 $${H}_{V}=\frac{1.8554P}{{d}^{2}}$$

where *P* is the load used in the test (GF) and* d* is the diagonal length of the microscopic indentation (μm).

To measure the fracture toughness, the same automatic Vickers microhardness tester (VIA-S) was used for testing. The load applied was 3 kgf, and the pressure holding was 10 s. The diagonal and crack lengths of the indentation were measured to calculate the fracture toughness, according to Eq. (2). All the samples were tested five times

2 $${{{K}_{IC}=0.018\left(\frac{E}{{H}_{V}}\right)}^{0.4}H}_{V}{a}^\frac{1}{2}{\left(\frac{l}{a}\right)}^{-0.5}$$

where *K*_*IC*_ denotes fracture toughness (MPa‧m^1/2^), *E* denotes the elastic modulus (GPa), *Hv* denotes the Vickers hardness (GPa), *a* denotes half of the diagonal length of the indentation, and* l* denotes the average crack length (nm)^14^.

In this paper, a UV–Vis spectrophotometer (UV-3900, Hitachi), the XploRA Laser Raman spectrometer (HORIBA Jobin Yvon), and a colorimeter (KONICA MINOLTA, CM-1700A) were used to test the optical properties of the ceramic samples.

## Results and discussion

### X-ray diffraction analysis

**Figure 1:**
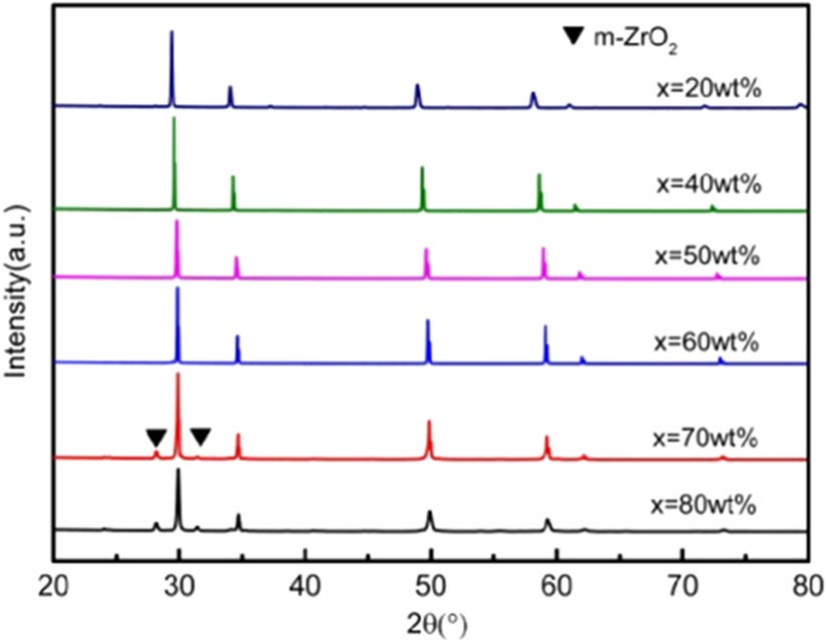
XRD patterns of ceramics sintered at 1500 °C with increasing 3YSZ fraction. *X* = wt%3YSZ (m-ZrO_2_: PDF#37-1484).

**Figure 2:**
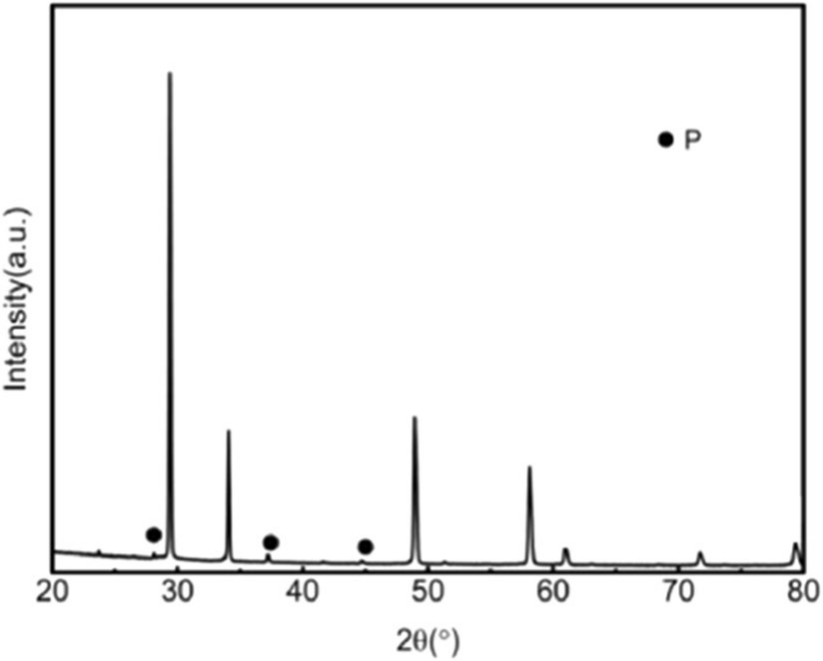
XRD patterns of the ceramic with 20wt%3YSZ sintered at 1500 °C (P: Pyrochlore structure: PDF#17-0458).

**Figure 3:**
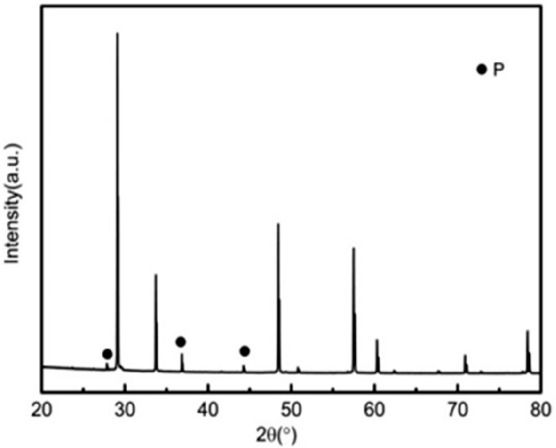
XRD patterns of the ceramic 0wt%3YSZ sintered at 1500 °C (P: Pyrochlore structure: PDF#17-0458).

**Figure 4:**
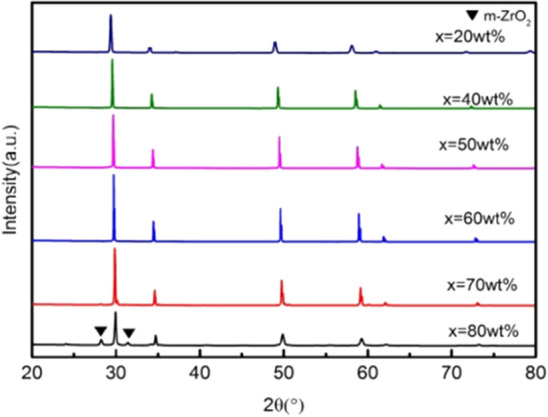
XRD patterns of ceramics sintered at 1450 °C with increasing 3YSZ fraction. *X* = wt%3YSZ (m-ZrO_2_: PDF#37-1484).

**Figure 5:**
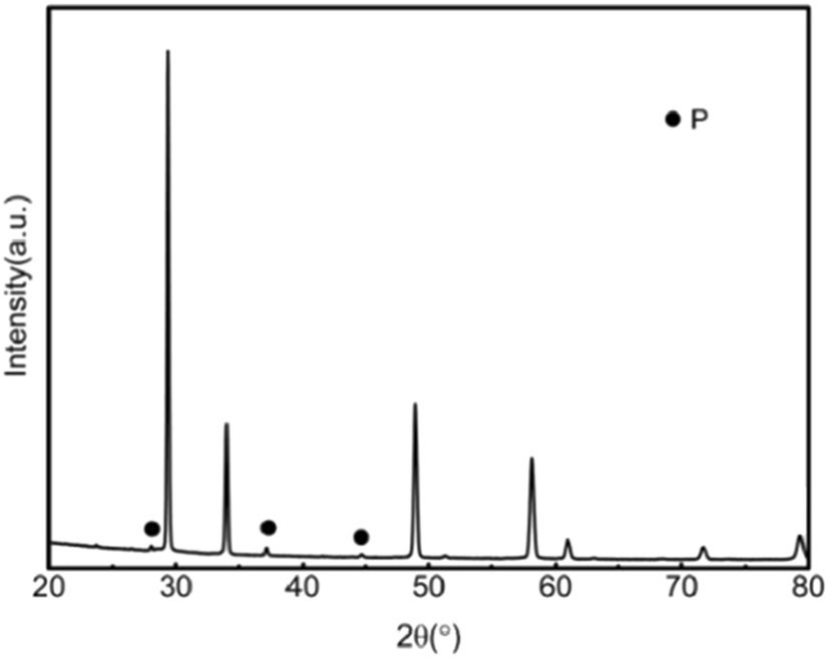
XRD patterns of the ceramic with 20wt%3YSZ sintered at 1450 °C (P: Pyrochlore structure: PDF#17-0458).

**Figure 6:**
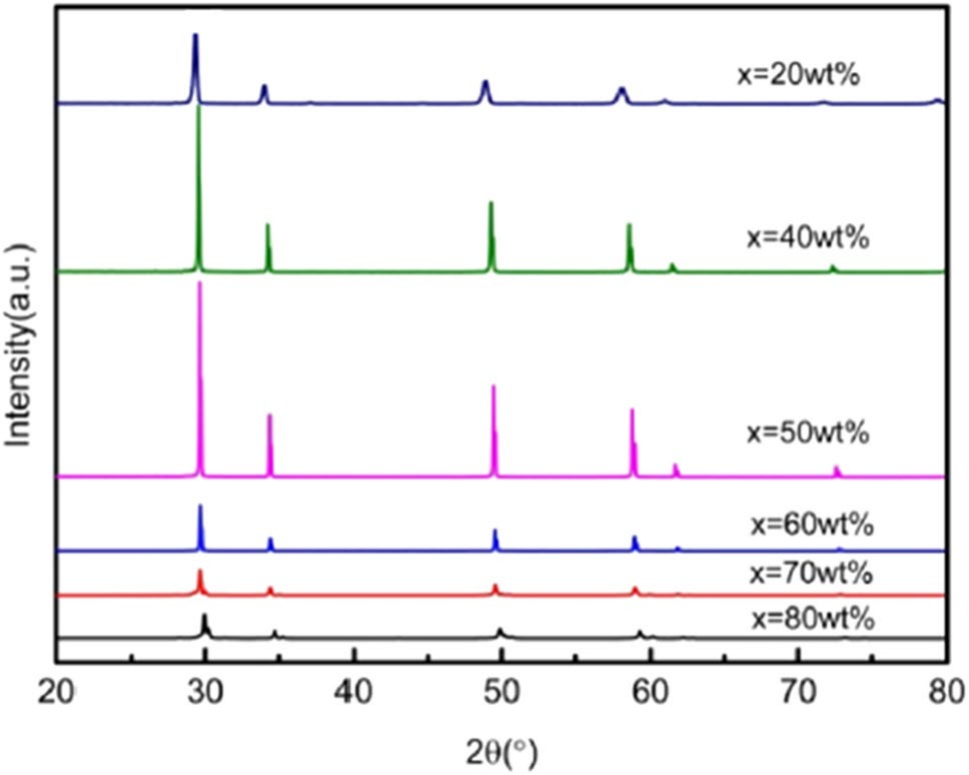
XRD patterns of ceramics sintered at 1400 °C with increasing 3YSZ fraction. *X* = wt%3YSZ.

**Figure 7:**
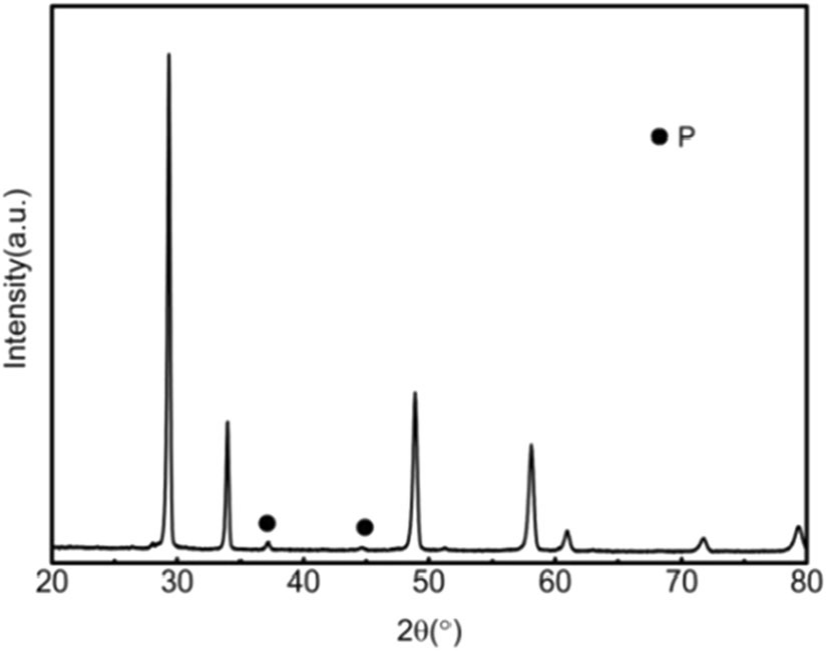
XRD patterns of the ceramic with 20wt%3YSZ sintered at 1400 °C (P: Pyrochlore structure: PDF#17-0458).

Figures 1, 2, 3, 4, 5, 6 and 7 shows the XRD pattern of the 3YSZ purple ceramic samples with different sintering temperatures and contents. It can be seen from Figs. 1, 2, 3, 4, 5, 6 and 7 despite different sintering temperatures and different composition of the mixture result in, the same crystalline phases, with similar XRD patterns.

As shown in Fig. 1, when the sintering temperature is 1500 °C, the main phase is the tetragonal phase of zirconia. When 3YSZ has mass fractions of 70% and 80%, the characteristic peaks of the monoclinic phase (− 111)m and (111)m appear at the diffraction angle 2θ of 28° and 31°, respectively. When 3YSZ is other content, there are no monoclinic (− 111)m and (111)m characteristic peaks at the diffraction angle 2θ of 28° and 31°, the others are the diffraction peaks of tetragonal zirconia. The presence of the tetragonal zirconia phase is good for the mechanical properties of ceramics. As shown in Figs. 2 and 3, when the mass fraction of 3YSZ is 20% and pure neodymium zirconate, the characteristic peaks (311), (331), (511) of the pyrochlore phase of neodymium zirconate when the diffraction angle 2θ is 27°, 37°, and 44.5° can be observed in the XRD pattern, respectively. This indicates that the tetragonal phase of zirconia and the chlorite phase of neodymium zirconate coexist. Since the characteristic peak of chlorite is not clear in the XRD spectrum, it cannot be seen in the higher content of 3YSZ, that is, a lower content of neodymium zirconate; however, it can be seen in the Raman spectrum.

As shown in Figs. 4 and 5, when the sintering temperature is 1450 °C, the main phase is also the tetragonal phase of zirconia. Different from the sintering temperature of 1500 °C, when 3YSZ has a mass fraction of 70%, no characteristic peaks of the monoclinic phase (− 111)m and (111)m are observed at the diffraction angle 2θ of 28°and 31°.

As shown in Figs. 6 and 7, when the sintering temperature is 1400 °C, the main phase is also the tetragonal phase of zirconia. Different from sintering temperature of 1500 °C and 1450 °C, when 3YSZ has mass fractions of 70% and 80%, the characteristic peaks of the monoclinic phase (− 111)m and (111)m do not appear at diffraction angle 2θ of 28°and 31°.

### Raman spectrogram analysis

**Figure 8:**
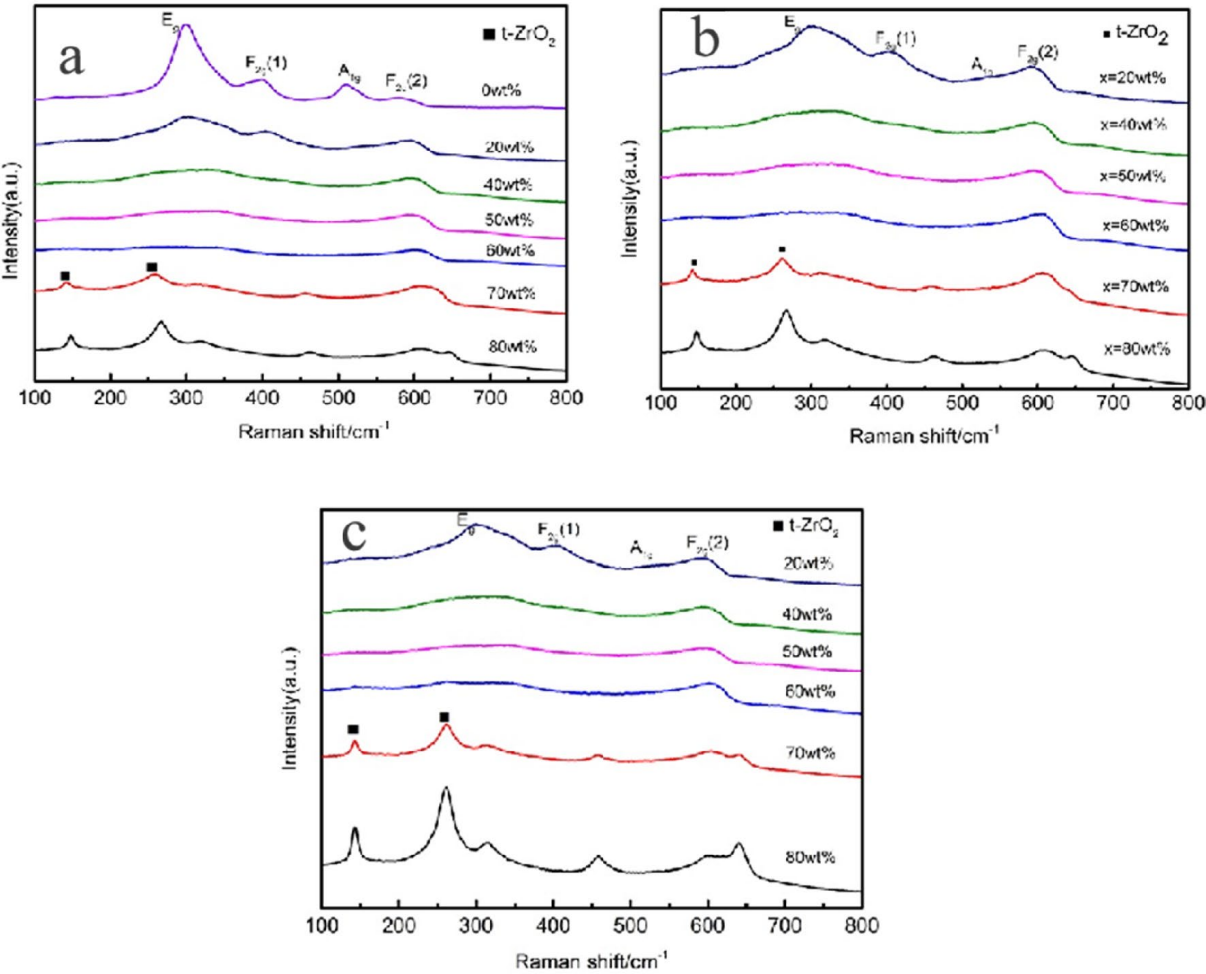
Raman spectra of ceramic samples containing various 3YSZ content and sintered (**a**) 1500 °C (**b**) 1450 °C (**c**) 1400 °C.

As shown from the three Raman spectra in Fig. 8, regardless of the sintering temperature, the Raman modes corresponding to pyrochlore can be seen as Eg mode at 300 cm^−1^, F2g mode at 400 cm^−1^ and 600 cm^−1^, A1g at 530 cm^−1^, and F2g mode at 750 cm^−1^.In Raman spectroscopy, the corresponding diffraction peak is not particularly obvious, and this situation has also been reported in relevant literature^15^. The ceramic samples prepared at the three sintering temperatures show the same trend of Raman spectra. In the Raman spectra of the prepared ceramic samples, it is found that with the increase of 3YSZ, the Raman peak becomes wider, and the intensity of the peak weakens. This phenomenon is due to the structural disorder^16^.

### Microstructure

**Figure 9:**
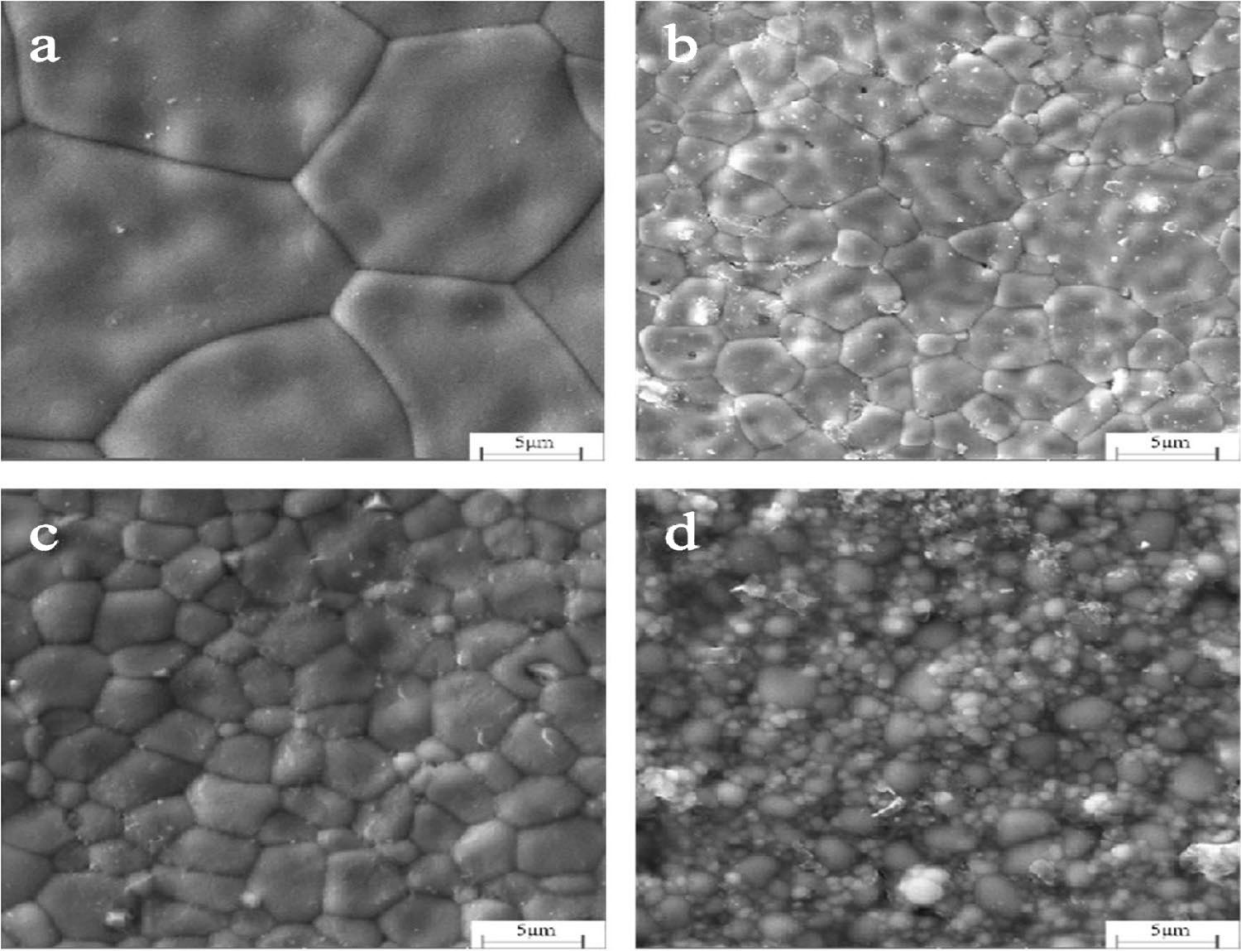
SEM images of *x*wt%3YSZ sintered at 1450 °C (**a**) *x* = 20, (**b**) *x* = 40, (**c**) *x* = 60, (**d**) *x* = 80.

**Figure 10:**
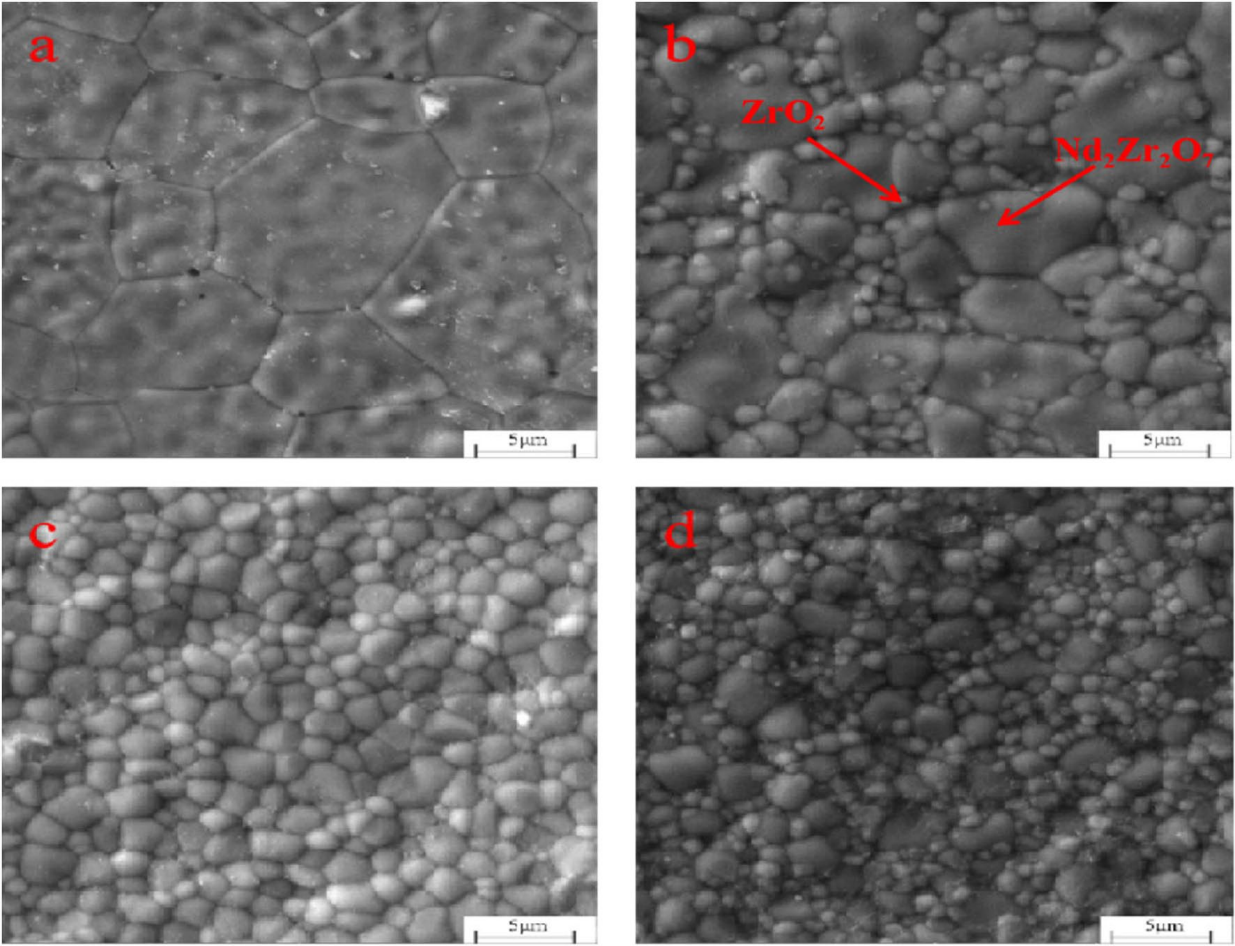
SEM images of 1400 °C* x*wt%3YSZ sintered at (**a**) *x* = 20, (**b**) *x* = 40, (**c**) *x* = 60, (**d**) *x* = 80.

Figure 9 and 10 show the surface of the various samples with different *x*wt% 3YSZ and different sintering temperatures. From the scanning electron microscopy (SEM), it can be see that all the samples have dense and uniform microstructure with polygonal grain and apparent, clean grain boundaries.

As shown in Fig. 9, when the sintering temperature is 1450 °C and the mass fraction of 3YSZ is 20%, it can be seen from the microscopic morphology that the grain size is still large. The grain size gradually decreased with the increase in the mass fraction of 3YSZ. When the mass fraction of 3YSZ reached 80%, the average size reached 0.8 μm. For such high amount of zirconia, the neodymium zirconate grain growth was hindered, resulting in improved mechanical properties of the ceramic, hindering the growth of the neodymium zirconate grain, resulting in a smaller neodymium zirconate grain. Therefore, its mechanical properties are improved.

As shown in Fig. 10, when the temperature drops to 1400 °C and 1450 °C, the grain size changes roughly with gradual decrease with the increase of the 3YSZ mass fraction. As show in Figs. 9 and 10, for 3YSZ with the same content of components, when the sintering temperature is different, it can be seen that the grain size decreases with the decrease of temperature.

**Figure 11:**
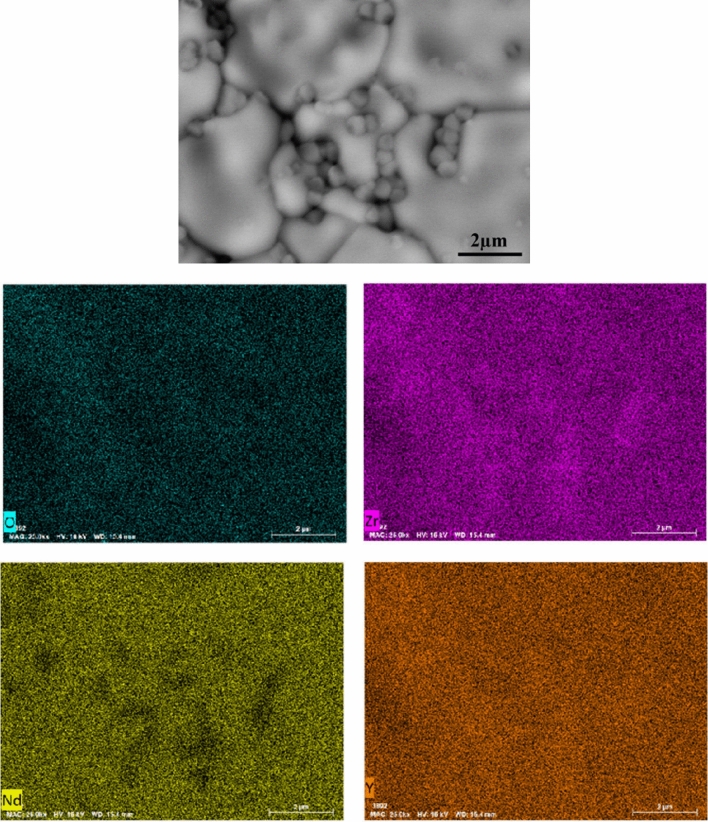
BSE and EDS images of sintered at 1400 °C 40wt%3YSZ.

As shown in Fig. 11, the above image shows BSE, and the following image shows EDS. By combining the two tests, we can see that the large grains are Nd_2_Zr_2_O_7_ and the small grains are zirconia, which are marked in Fig. 10b.

### Optical properties

#### Chroma analysis

**Figure 12:**
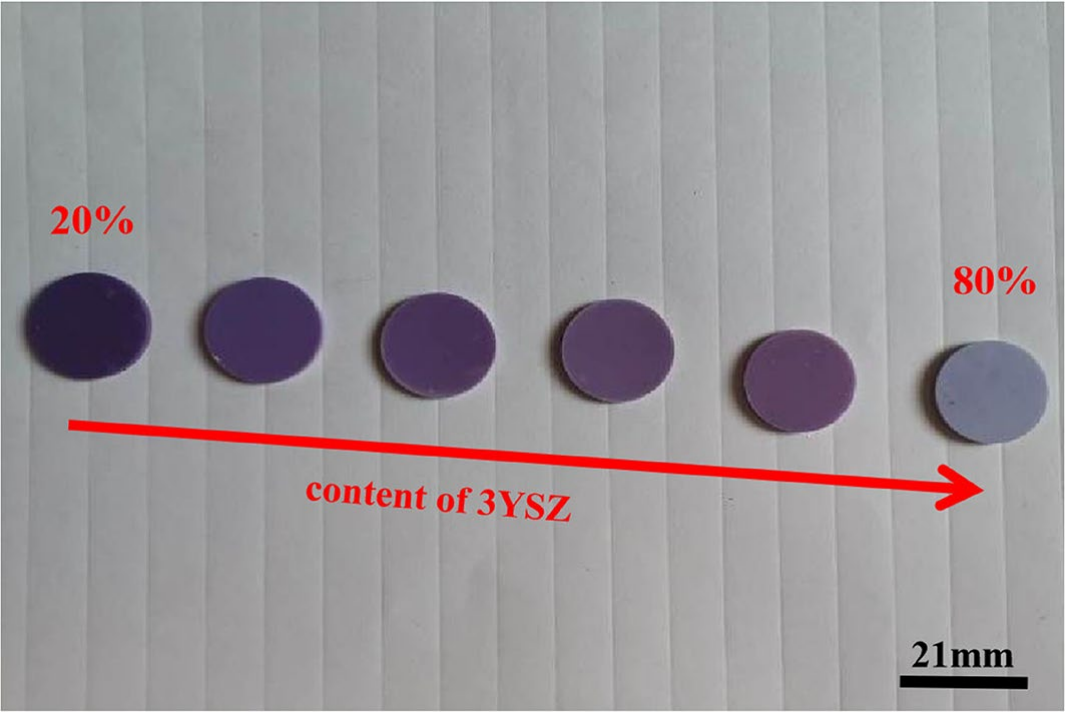
Picture of ceramic samples sintered at 1400 °C.

As shown in Fig. 12, for a fixed sintering temperature of 1400 °C, the purple degree of the ceramic sample decreases with the increase of the 3YSZ mass fraction.

Tables 1, 2 and 3 present the color (chromaticity value) of the ceramic samples. It can be seen that for the same sintering temperature, with the increase of 3YSZ mass fraction, the brightness value (L*) of all ceramic samples becomes larger overall. Due to the weakened absorption of visible light by the ceramic sample, the color becomes shallow. For ceramic specimens with the same 3YSZ mass fraction, from the point of view of the chromaticity value, the lower the temperature, the smaller the brightness value (L*). However, the difference in the chromaticity value (L*) is little, indicating that the temperature has minor effect on the color as compared to the composition.

**Table 1:** Chromaticity values of ceramics sintered sample at 1500 °C.

3YSZ content Chroma value	20%	40%	50%	60%	70%	80%
a*	12.84 ± 0.01	12.51 ± 0.03	12.25 ± 0.03	11.96 ± 0.02	11.65 ± 0.04	9.50 ± 0.01
b*	11.81 ± 0.02	14.75 ± 0.03	14.22 ± 0.01	13.62 ± 0.01	12.10 ± 0.02	13.10 ± 0.03
L*	50.39 ± 0.01	60.97 ± 0.03	59.56 ± 0.03	68.42 ± 0.02	65.23 ± 0.02	68.44 ± 0.04

**Table 2:** Chromaticity values of ceramics sintered sample at 1450 °C.

3YSZ content Chroma value	20%	40%	50%	60%	70%	80%
a*	9.70 ± 0.01	10.40 ± 0.02	11.14 ± 0.03	10.31 ± 0.01	10.20 ± 0.02	8.37 ± 0.01
b*	11.95 ± 0.03	15.95 ± 0.02	15.08 ± 0.03	15.72 ± 0.03	14.97 ± 0.03	15.01 ± 0.03
L*	49.27 ± 0.03	63.98 ± 0.01	66.26 ± 0.02	67.44 ± 0.02	64.06 ± 0.04	67.37 ± 0.04

**Table 3:** Chromaticity values of ceramics sintered sample at 1400 °C.

3YSZ content Chroma value	20%	40%	50%	60%	70%	80%
a*	9.82 ± 0.03	9.32 ± 0.01	9.61 ± 0.01	8.34 ± 0.03	8.64 ± 0.01	8.94 ± 0.02
b*	10.73 ± 0.01	14.48 ± 0.03	15.01 ± 0.02	15.04 ± 0.03	14.81 ± 0.01	15.22 ± 0.03
L*	48.01 ± 0.02	62.42 ± 0.01	64.65 ± 0.03	67.43 ± 0.03	67.56 ± 0.02	68.23 ± 0.04

#### UV reflectance spectroscopy analysis

**Figure 13:**
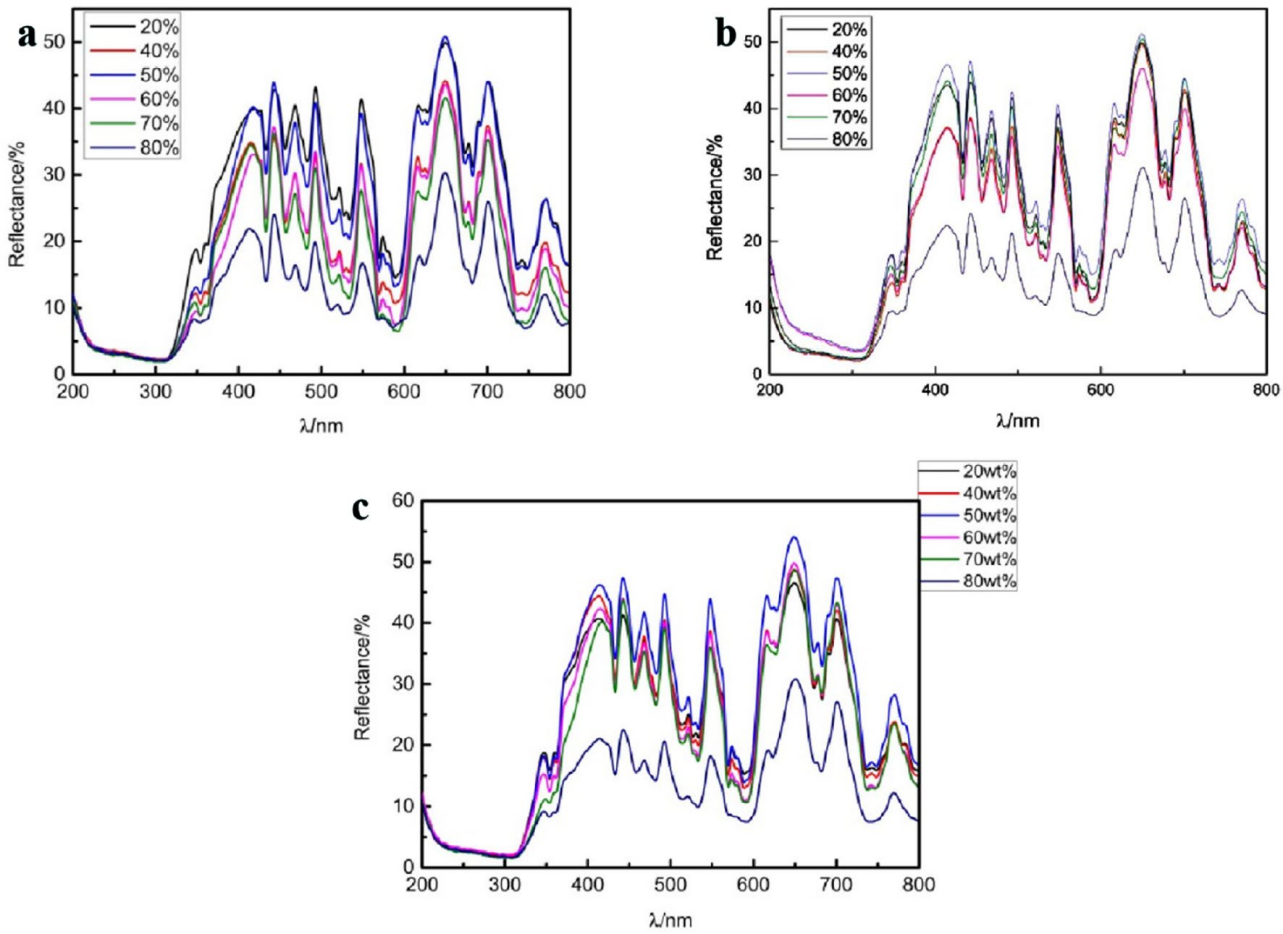
UV reflectance spectra of purple ceramics sintered at different temperatures. (**a**) 1500 °C (**b**) 1450 °C (**c**) 1400 °C.

Figure 13 shows the UV reflectance spectra of the prepared ceramic samples. It can be seen that the trend of change is roughly the same for all sintering temperatures and compositions. For the same compositions, the reflectivity at all sintering temperatures at the same wavelength remains basically unchanged, indicating that the temperature has little effect on it. For the same sintering temperature, it can be found instead that in the range of 400 ~ 435-nm violet light wavelength, purple light reflection increases, and the color purple is deepening. Therefore, as the content of 3YSZ decreases, the ceramic reflectivity gradually increases.

### Mechanical properties

#### Vickers hardness

**Figure 14:**
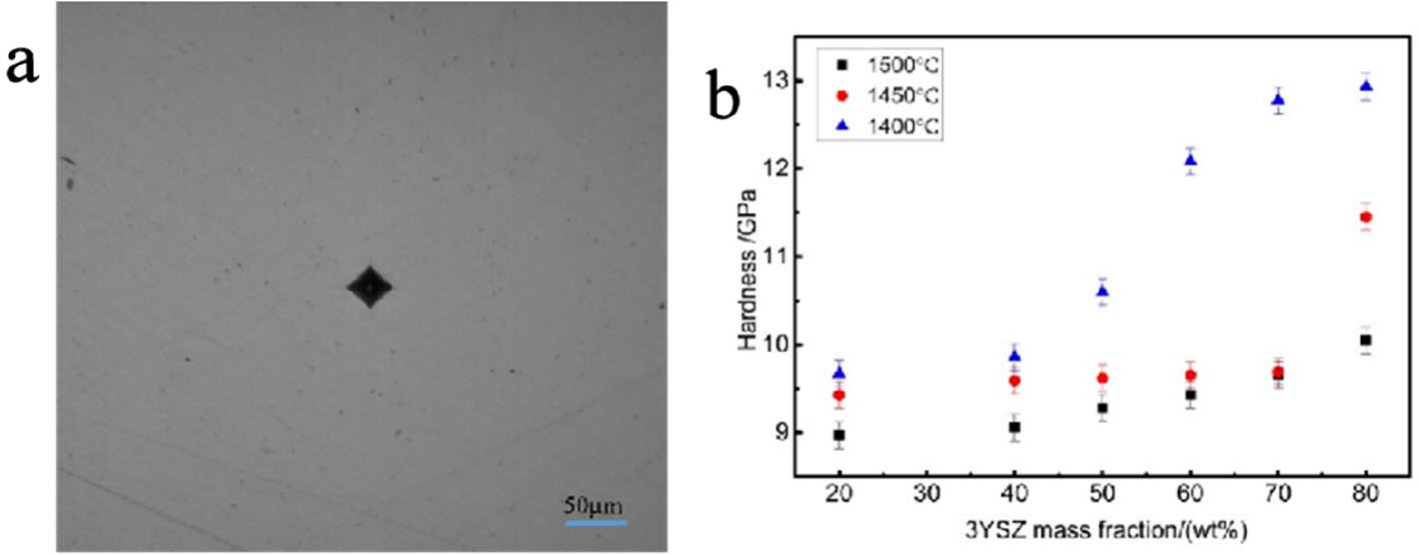
(**a**) Indentation of ceramic samples with a 3YSZ content of 80% sintered at 1400 °C (Load is 2 Kgf). (**b**) Vickers hardness values of ceramic samples sintered at various temperatures and with different 3YSZ fractions.

From Fig. 14 it can be seen that, compared with pure neodymium zirconate, the Vickers hardness improved regardless the mass fraction of ZrO_2_. When the content of 3YSZ is 80% and the sintering temperature is 1400 °C, the maximum Vickers hardness is 12.93 GPa, which is nearly 88% higher than that of pure neodymium zirconate, 6.86 GPa. This is due to the refined grain size of the ceramic. For the same sintering temperature, the Vickers hardness value gradually increases with the increase in the 3YSZ mass fraction. For the same 3YSZ content, the Vickers hardness value increases as the sintering temperature decreases. Based on the analysis of the field emission scanning electron microscopy images, for the same sintering temperature, the higher the mass fraction of 3YSZ, the smaller the grain size, and the higher is the hardness. For the same 3YSZ content, the lower the temperature, the smaller the grain size, and the greater is the ability to resist deformation and the corresponding hardness.

#### Fracture toughness

**Figure 15:**
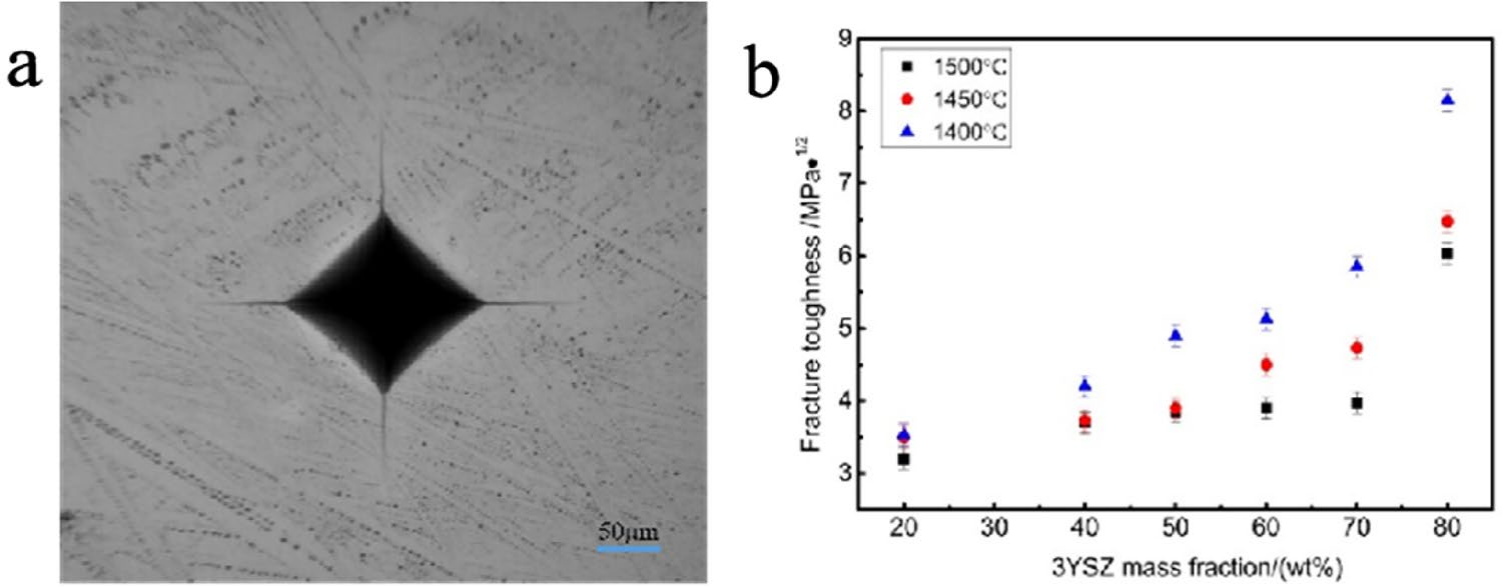
(**a**) Indentation of ceramic samples with a 3YSZ content of 80% sintered at 1400 °C (Load is 3 Kgf). (**b**) fracture toughness values of ceramic samples sintered at various temperatures and with different 3YSZ fractions.

As shown in Fig. 15, compared with pure neodymium zirconate, the fracture toughness improved, regardless the mass fraction of ZrO_2_ additions. When the 3YSZ content was 80% and the sintering temperature is 1400 °C, the fracture toughness is the maximum, 8.15 MPa‧m^1/2^, which is nearly twice higher than pure neodymium zirconate, 2.57 MPa‧m^1/2^.

**Figure 16:**
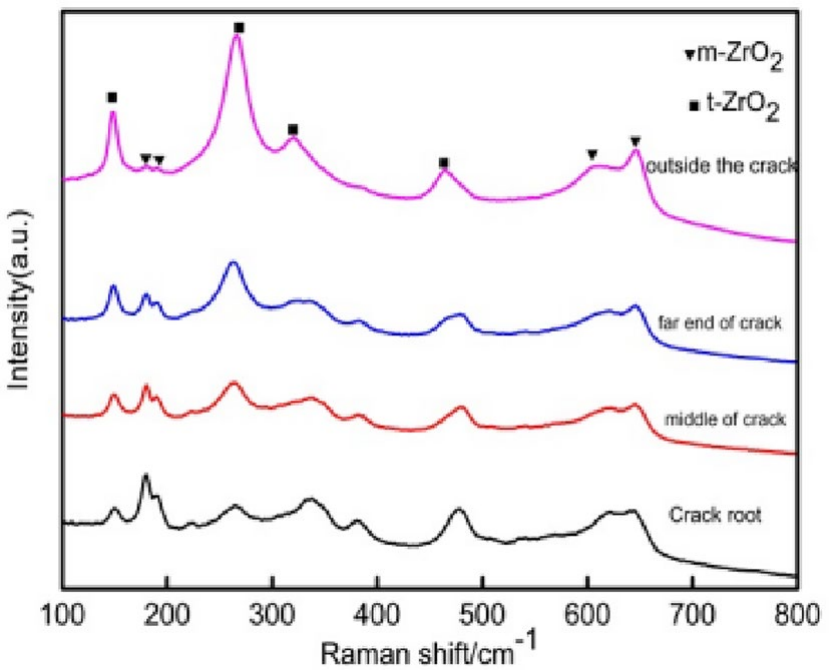
Raman spectra at the indentation of the ceramic sample sintered at 1400 °C with 3YSZ amount equal to 80%.

As shown in Figs. 9 and 10, the addition of the second phase improves the density of the ceramic sample and also makes the grain fines, making also the grain size of neodymium zirconate much smaller. In addition, it also hinders the crack propagation, thus improving the fracture toughness of ceramic materials. To explore whether the toughening mechanism in NZO is due to phase change toughening, Fig. 16, shows that the monoclinic phase (m-ZrO_2_) of zirconia appears in both 178 cm^−1^ and 188 cm^−1^ of the tested Raman spectra of the 3YSZ quality score is 80% sample, indicating that a martensitic transformation from t-ZrO_2_ to m-ZrO_2_ has occurred.

## Conclusions

Purple ceramics can be prepared by mixtures of neodymium zirconate and yttria-stabilized-zirconia (3YSZ) and sintered in the 1400–1500 °C temperature range of resulting in a broad range shades to be possibly used in smart wearables.

The results of UV reflectance spectroscopy analysis show that with the increase in the 3YSZ content, the absorption of visible light by the ceramic sample decreases, and the brightness value of the ceramic sample increases.

The addition of 3YSZ to neodymium zirconate results in refined grains, thus improving its mechanical properties: the Vickers hardness increased to 12.93 GPa for for 80wt% addition of 3YSZ, which is an increase of nearly 88% compared to pure neodymium zirconate; fracture toughness 8.15 MPa‧m^1/2^, which is nearly twice as high compared to pure neodymium zirconate due to phase change toughening.

## Data Availability

All data generated or analysed during this study are included in this published article (and its Supplementary Information files).
