# Author Correction: Study on the preparation and mechanical properties of purple ceramics

**DOI:** 10.1038/s41598-023-38494-y

**Published:** 2023-07-27

**Authors:** Lihe Wang, Yonghui Wang, Qingchun Wang, Yuwei Ma, Fei Ruan, Yonghe Zhang, Haodong Lv, Qiang Jing, Jinxiao Bao

**Affiliations:** Inner Mongolia Key Laboratory of Advanced Ceramics and Device, School of Materials and Metallurgy, Inner Mongolia University of Science and Technology, Baotou, 014010 Inner Mongolia China

Correction to: *Scientific Reports* 10.1038/s41598-023-35957-0, published on 30 May 2023

The original version of this Article contained errors in Figures 1, 2, 4, 5, 6, 7 and the accompanying legends, where the labels of the tetragonal phase were incorrect. The original Figures 1, 2, 4, 5, 6, 7 and their legends appear below.

**Figure 1:**
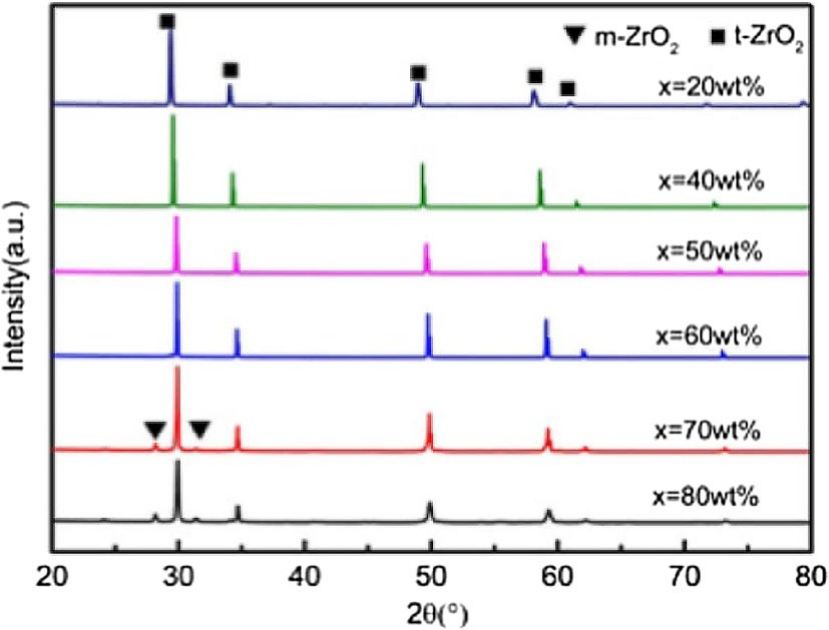
XRD patterns of ceramics sintered at 1500 °C with increasing 3YSZ fraction. *X* = wt%3YSZ. (m-ZrO_2_: PDF#37-1484; t-ZrO_2_: PDF#50-1089).

**Figure 2:**
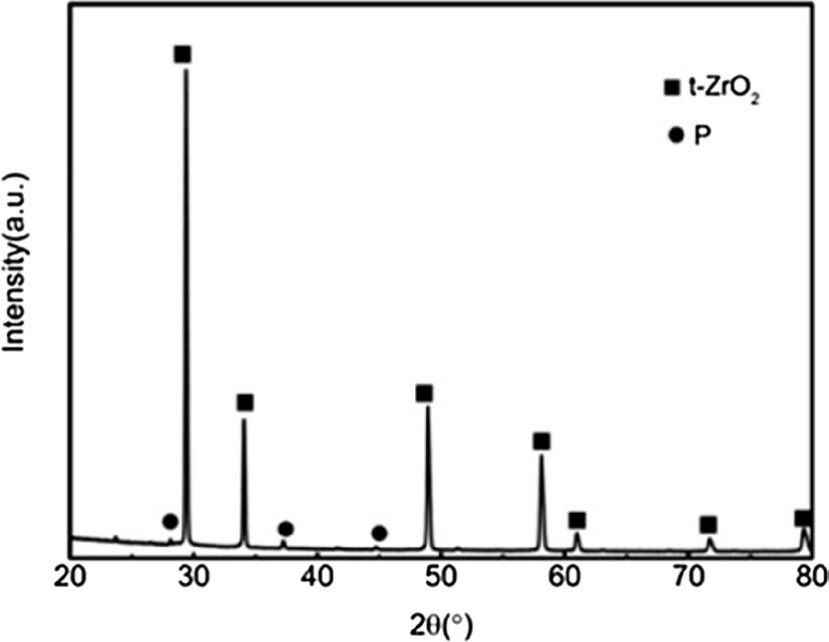
XRD patterns of the ceramic with 20wt%3YSZ sintered at 1500 °C. (t-ZrO_2_: PDF#50-1089; P: Pyrochlore structure : PDF#17-0458).

**Figure 4:**
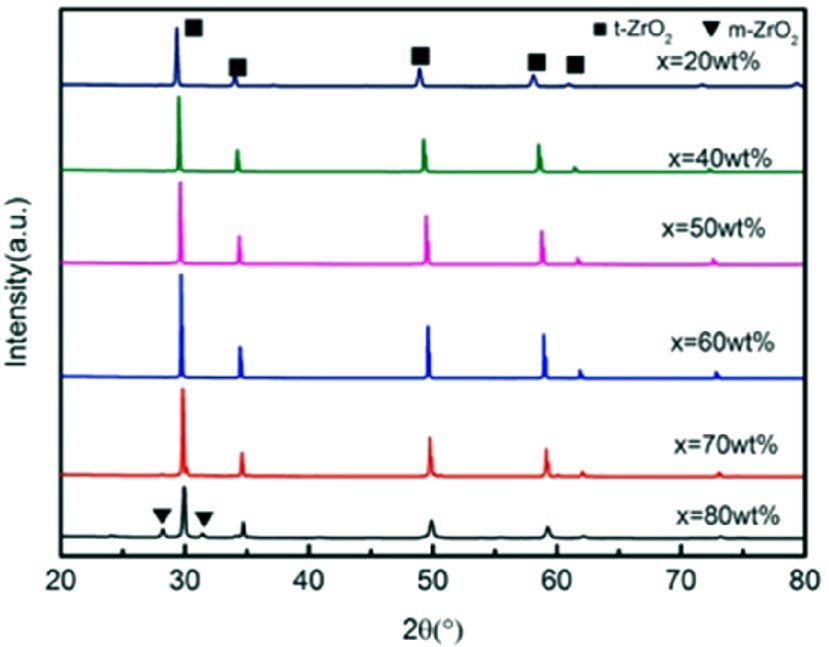
XRD patterns of ceramics sintered at 1450 °C with increasing 3YSZ fraction. *X* = wt%3YSZ. (m-ZrO_2_: PDF#37-1484; t-ZrO_2_: PDF#50-1089).

**Figure 5:**
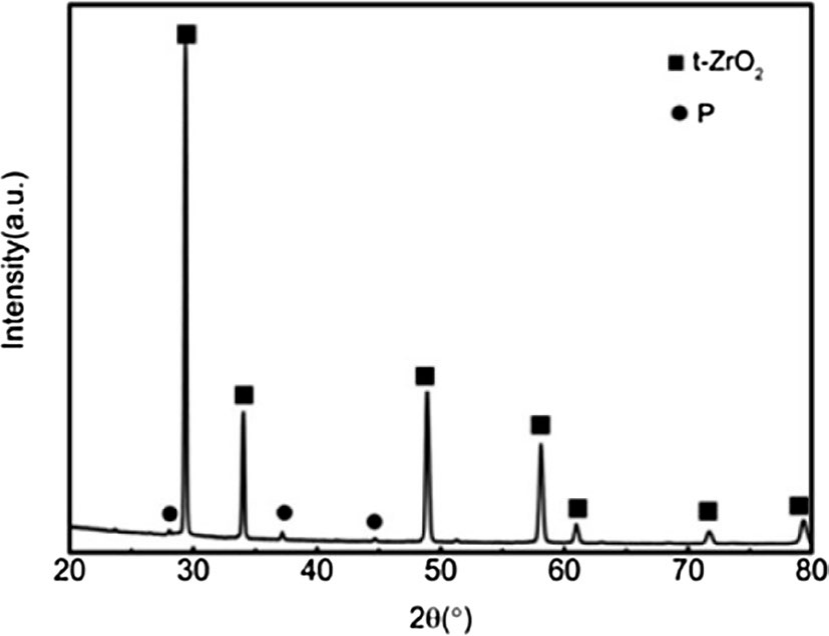
XRD patterns of the ceramic with 20wt%3YSZ sintered at 1450 °C. (t-ZrO_2_: PDF#50-1089; P: Pyrochlore structure : PDF#17-0458).

**Figure 6:**
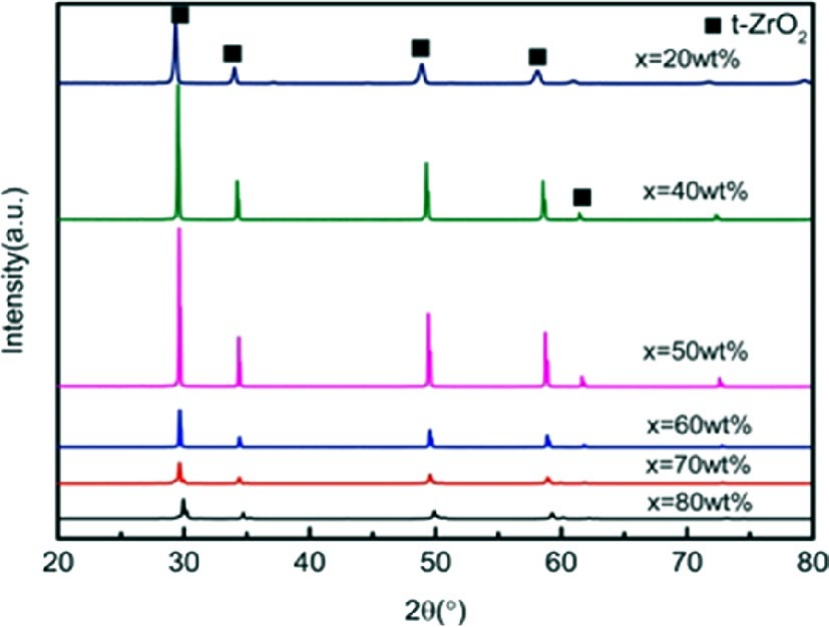
XRD patterns of ceramics sintered at 1400 °C with increasing 3YSZ. fraction *X* = wt%3YSZ. (m-ZrO_2_: PDF#37-1484; t-ZrO_2_: PDF#50-1089).

**Figure 7:**
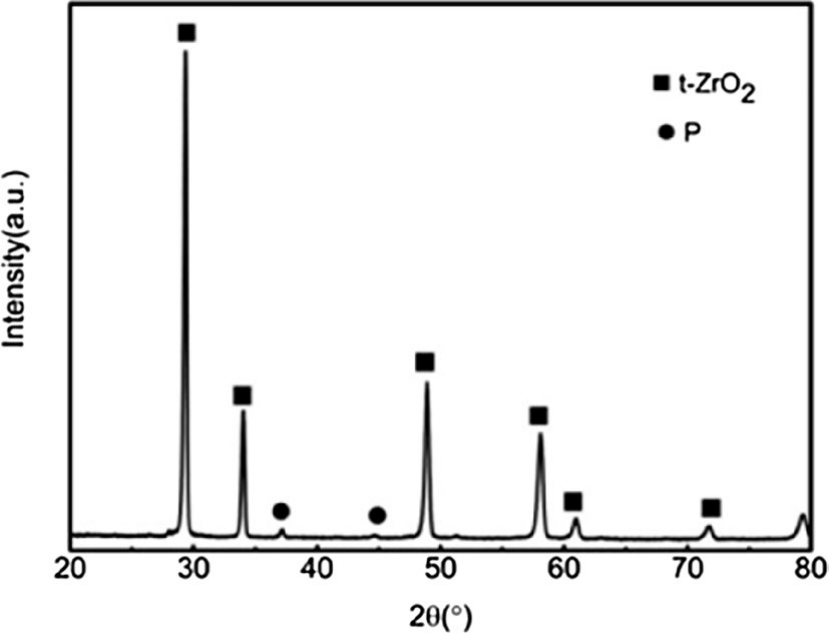
XRD patterns of the ceramic with 20wt%3YSZ sintered at 1400 °C. (t-ZrO_2_: PDF#50-1089; P: Pyrochlore structure : PDF#17-0458).

The original Article has been corrected.

